# Triple antiplatelet therapy for preventing vascular events: a systematic review and meta-analysis

**DOI:** 10.1186/1741-7015-8-36

**Published:** 2010-06-16

**Authors:** Chamila Geeganage, Robert Wilcox, Philip MW Bath

**Affiliations:** 1 Stroke Trials Unit, Institute of Neuroscience, Division of Stroke, University of Nottingham, City Hospital campus, Nottingham NG5 1PB, UK; 2 Division of Cardiovascular Medicine, D Floor, South Block, University Hospital, Queen's Medical Centre, Nottingham NG7 2UH, UK

## Abstract

**Background:**

Dual antiplatelet therapy is usually superior to mono therapy in preventing recurrent vascular events (VEs). This systematic review assesses the safety and efficacy of triple antiplatelet therapy in comparison with dual therapy in reducing recurrent vascular events.

**Methods:**

Completed randomized controlled trials investigating the effect of triple versus dual antiplatelet therapy in patients with ischaemic heart disease (IHD), cerebrovascular disease or peripheral vascular disease were identified using electronic bibliographic searches. Data were extracted on composite VEs, myocardial infarction (MI), stroke, death and bleeding and analysed with Cochrane Review Manager software. Odds ratios (OR) and 95% confidence intervals (CI) were calculated using random effects models.

**Results:**

Twenty-five completed randomized trials (17,383 patients with IHD) were included which involving the use of intravenous (iv) GP IIb/IIIa inhibitors (abciximab, eptifibatide, tirofiban), aspirin, clopidogrel and/or cilostazol. In comparison with aspirin-based therapy, triple therapy using an intravenous GP IIb/IIIa inhibitor significantly reduced composite VEs and MI in patients with non-ST elevation acute coronary syndromes (NSTE-ACS) (VE: OR 0.69, 95% CI 0.55-0.86; MI: OR 0.70, 95% CI 0.56-0.88) and ST elevation myocardial infarction (STEMI) (VE: OR 0.39, 95% CI 0.30-0.51; MI: OR 0.26, 95% CI 0.17-0.38). A significant reduction in death was also noted in STEMI patients treated with GP IIb/IIIa based triple therapy (OR 0.69, 95% CI 0.49-0.99). Increased minor bleeding was noted in STEMI and elective percutaneous coronary intervention (PCI) patients treated with GP IIb/IIIa based triple therapy. Stroke events were too infrequent for us to be able to identify meaningful trends and no data were available for patients recruited into trials on the basis of stroke or peripheral vascular disease.

**Conclusions:**

Triple antiplatelet therapy based on iv GPIIb/IIIa inhibitors was more effective than aspirin-based dual therapy in reducing VEs in patients with acute coronary syndromes (STEMI and NSTEMI). Minor bleeding was increased among STEMI and elective PCI patients treated with a GP IIb/IIIa based triple therapy. In patients undergoing elective PCI, triple therapy had no beneficial effect and was associated with an 80% increase in transfusions and an eightfold increase in thrombocytopenia. Insufficient data exist for patients with prior ischaemic stroke and peripheral vascular disease and further research is needed in these groups of patients.

## Background

Platelets contribute to the pathogenesis of different vascular syndromes including myocardial infarction (MI), ischaemic stroke and peripheral artery disease. Antiplatelet therapy offers partial prevention of these events[1-4]. The current therapeutic strategies for inhibiting platelets include: inhibition of cyclooxygenase (for example, aspirin [5]); inhibition of phosphodiesterases III and V and uptake by red cells of adenosine (for example, cilostazol, dipyridamole); blockade of the platelet ADP P2Y12 receptor (for example, ticlopidine, clopidogrel, prasugrel); blockade of glycoprotein IIb/IIIa receptors (which prevents fibrinogen binding); and increasing nitric oxide levels (for example, triflusal). While most antiplatelet agents are usually given orally, glycoprotein IIb/IIIa receptor antagonists can be given intravenously (for example, abciximab, eptifibatide, tirofiban) or orally (for example, lotrafiban, orbofiban, sibrafiban, xemilofiban). However, oral IIb/IIIa receptor antagonists have been abandoned due to an increase in death in several trials[6].

Individual antiplatelet agents reduce recurrent events by 15%-20%, as seen with aspirin and dipyridamole [7,8] and from indirect comparisons for clopidogrel, triflusal and cilostazol[9-11]. These drugs have different mechanisms of action so their combination is likely to be additive and more effective in reducing vascular events than monotherapy, a hypothesis confirmed for aspirin and clopidogrel [12-15] and aspirin and dipyridamole [8,16]. As a result, guidelines now recommend dual combinations for patients with non-ST elevation with acute coronary syndromes (NSTE-ACS), ST elevation with myocardial infarction (STEMI), percutaneous coronary infarction (PCI) and ischaemic stroke/transient ischaemic attack (TIA) [17-20]. However, the combination of aspirin and clopidogrel is not recommended for long-term prophylaxis (> 12 months) against stroke because of excess bleeding, as seen in MATCH and CHARISMA[21,22]. Further, in the setting of high risk NSTE-ACS (patients having elevated troponins, ST depression, or diabetes) addition of eptifibatide or tirofiban to oral antiplatelet agents is recommended for initial early treatment (class II, a level A)[19,20]. Addition of abciximab to aspirin and clopidogrel is also recommended in both NSTE-ACS and STEMI patients undergoing PCI (for NSTE-ACS class 11 level B)[19,20]. However, in patients with recent stroke, the PRoFESS trial found that the combination of aspirin plus extended release dipyridamole versus clopidogrel had a comparable effect on secondary stroke prevention[23]. However, the benefit of combined antiplatelet therapy during high risk acute ischaemic stroke/TIAs is still unknown.

If two agents are superior to one, then three might be even better providing that bleeding does not become a limiting factor. Several randomized trials have compared triple antiplatelet therapy with dual therapy and we have assessed these in a systematic review involving patients with ischaemic vascular diseases.

## Methods

### Ethics

No ethical approval was required for this study.

### Search strategy

Completed randomized controlled trials that investigated the effect of triple antiplatelet versus dual antiplatelet therapy in the prevention of vascular events in patients with ischaemic vascular diseases [stroke/TIA, ischaemic heart disease (IHD), peripheral vascular disease (PVD)] were sought with searches (October 2009) of electronic databases including Cochrane Library (issue 4 2009), EMBASE, MEDLINE and Science Citation Index (ISI Web of Science). Separate search strategies were developed for each database using the following keywords: 'triple antiplatelet therapy', 'aggressive antiplatelet therapy', 'combined antiplatelet therapy', 'antiplatelet therapy', 'aspirin', 'dipyridamole', 'clopidogrel', 'ticlopidine', 'prasugrel' 'cilostazol', 'triflusal', 'abciximab', 'tirofiban', 'eptifibatide', 'ST elevation myocardial infarction', 'non-ST elevation acute coronary syndrome', 'stroke', 'transient ischaemic attack', 'acute limb ischaemia', 'peripheral arterial/vascular disease', 'vascular events', 'randomized controlled trials' and 'controlled clinical trials'. Reference lists of earlier reviews [24,25] and identified trial publications were also checked for additional trials. Where duplicate publications were identified, data from the primary report were used. Publications could be in any language.

### Selection

Completed randomized, placebo or open-label, controlled trials were included where adult patients who were at high risk of ischaemic VEs were enrolled: NSTE-ACS; STEMI; PVD; acute ischaemic stroke; TIA; and previous MI/or coronary artery disease (secondary prevention), including elective PCI; previous stroke and/or TIA; and peripheral arterial disease. Patients had to have been treated with triple antiplatelet therapy versus dual antiplatelet therapy. Trials where only a sub-set of patients were offered triple therapy were not included.

### Validity assessment

Trials were identified based on the inclusion/exclusion criteria discussed above. Methodological quality of the trials was assessed in relation to randomization and concealment of allocation. A quality scale was used to assess the trials: (A) true randomization and allocation concealed; and (B) process of randomisation not given and concealment of allocation unclear. This approach is recommended by the Cochrane collaboration[26].

### Data abstraction

Two reviewers identified and assessed published trials. One author resolved disagreements on studies by discussion (PB).

### Study characteristics

The following information were extracted by treatment group: (i) treatment, including type and route of antiplatelet administration, treatment window, length of treatment, and follow up period; (ii) number of patients; and (iii) outcome, this encompassing: composite vascular events (non fatal stroke, non-fatal myocardial infarction or death), myocardial infarction, ischaemic stroke, death, major bleeding, intracranial bleeding, minor bleeding, blood transfusions and thrombocytopenia. Outcome events were based on the definitions used in the individual trial publications. The primary outcome comprised composite vascular events; other events were regarded as secondary outcomes.

### Quantitative data synthesis

Data were entered into and analysed using the Cochrane Collaboration Review Manager software (version 4.2). Data were analysed separately by indication (NSTE-ACS, STEMI, PCI, ischaemic stroke, TIA, PVD and indicator drug in addition to the overall assessment of each vascular outcome. Odds ratios (OR) and 95% confidence intervals (CI) were calculated; random-effects models were used since heterogeneity was expected among the trials taking account that different antiplatelet agents and patient populations were being studied. Heterogeneity was calculated using the Chi-squared and I^2 ^statistics. An OR < 1 suggests a beneficial effect whilst an OR > 1 suggest a detrimental drug effect. Absolute event rates for composite VE (primary outcome) and bleeding were calculated. The event rates for patients receiving triple therapy was calculated from the relative risk and control event rate for each outcome because the unequal randomization in some trials makes exact rates in the treatment group unreliable ('Simpson paradox'). Egger's test and Beggs funnel-plot were performed to assess any publication bias in included trials[27].

## Results

### Trial flow/flow of included studies

**Figure 1:**
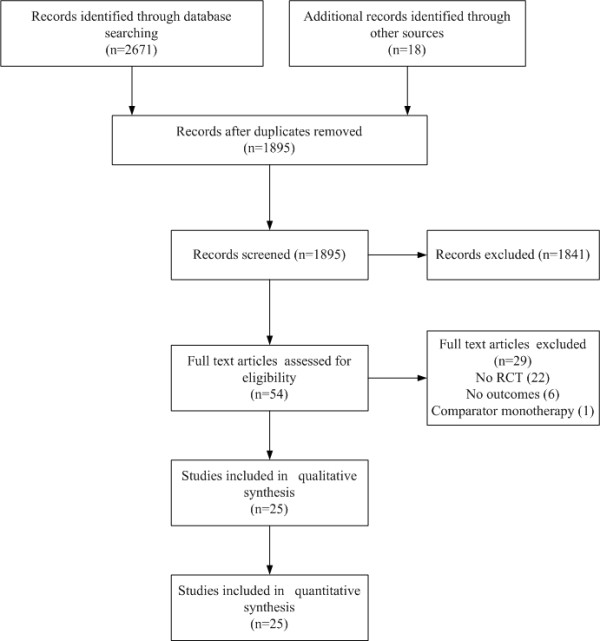
**Search process for relevant studies**.

Twenty-five completed randomized trials fulfilled the inclusion criteria (Table 1) and included 17,383 patients (Figure 1). Seven trials (771 patients) were excluded (Figure 1), mostly because they did not provide relevant outcome data or did not have a control group; an eighth trial is ongoing.

**Table 1:** Included randomized controlled trials.

				Control group							
Study	N	Age	Male (%)	Asp	TP	GP	Triple group	Heparin	Length of treatment	Length of follow-up (months)	Quality*
NSTE-ACS											
ADVANCE[41]	202	69	68	√	√		Tirofiban	√	48 h	6	A
ELISA-2[42]	328	64	71	√	√		Tirofiban	**-**	12 h	1	A
ISAR-REACT-2[43]	2,022	66	75	√	√		Abciximab	√	12 h	1	A
PROTECT-TIMI-30[44]	857	60	67	√	√		Eptifibatide	√	24 h	48 h	A
STEMI											
ADMIRAL[45]	300	61	82	√	√		Abciximab	√	12 h	6	A
ACE[46]	400	64	77	√	√		Abciximab	√	12 h	6	A
CADILLAC [47]	2082	60	73	√	√		Abciximab	√	12 h	6	B
Ernst/1[48]	60	61	74	√	√		Abciximab	√	12 h	1	B
Ernst/2[48]	60	60	82	√	√		Tirofiban	√	12 h	1	B
Ernst/3[48]	59	61	76	√	√		Tirofiban high dose	√	12 h	1	B
ISAR 2[30]	401	61	76	√	√		Abciximab	√	12 h	1	B
Neumann[31]	200	60	77	√	√		Abciximab	√	12 h	1	B
Petronio[49]	31	57	87	√	√		Abciximab	√	12 h	1	B
Kim[50]	60	63	70	√	√		Cilostazol	√	1 month	1	B
Elective PCI											
Claeys[51]	200	67	70	√	√		Abciximab	√	12 h	6	B
EPISTENT[52]	1,603	59	75	√	√		Abciximab	√	13 h	1	A
ESPRIT[35]	2,064	62	73	√	√		Eptifibatide	√	24 h	1	B
ISAR-REACT[53]	2,159	66	77	√	√		Abciximab	√	12 h	1	A
ISAR-SMART-2[54]	502	66	73	√	√		Abciximab	√	12 h	12	A
ISAR-SWEET[55]	701	68	75	√	√		Abciximab	√	12 h	12	A
TOPSTAR[56]	96	65	75	√	√		tirofiban	√	42 h	9	A
MR PCI/1[57]	60	61	85	√	**-**	√	Clopidogrel	√	30 days	1	A
MR PCI/2[57]	60	56	85	√	**-**	√	Clopidogrel	√	30 days	1	A
DECLARE-Long[58]	500	61	64	√	√		Cilostazol	**-**	6 months	6	A
DECLARE-DIABETES[59]	400	61	58	√	√		Cilostazol	**-**	6 months	6	A
Min[60]	59	62	66	√	√		Cilostazol	√	1 month	6	A
CREST[61]	705	60	74	√	√		Cilostazol	**-**	6 months	6	A
Han[62]	1212	60	73	√	√		Cilostazol	**-**	6 months	12	A

*Quality scale: A, true randomization and allocation concealed; B, process of randomization not given and concealment of allocation unclear.

Asp, aspirin; GP, glycoprotein IIb/IIIa receptor antagonists (abciximab, tirofiban, eptifibatide); MI, myocardial infarction; NSTEACS, non-ST elevation acute coronary syndrome; PCI, percutaneous coronary interventions; TP, thienopyridine (clopidogrel, ticlopidine).

### Study characteristics

The included trials studied three patient populations: NSTE-ACS (four trials), STEMI (8) and elective PCI (13) (Table 1). No studies in patients with previous stroke or PVD were identified. Each of the 25 trials was randomized and 17 had concealed allocation; allocation concealment was unclear in eight studies[28-35]. All trials compared triple antiplatelet therapy with dual therapy (Table 1). Most trials used an intravenous GP IIb/IIIa receptor antagonist as the additional antiplatelet agent (no studies used an oral GP IIb/IIIa receptor antagonist, presumably because of hazard with these agents [6]); the remaining trials used clopidogrel or cilostazol as the extra agents. No triple therapy trials involved dipyridamole, triflusal or prasugrel. Twenty trials gave concomitant heparin to both treatment groups. Hence, the comparison of triple versus conventional antiplatelet therapy was not confounded (Table 1). No evidence of publication bias using Egger's test (*P *for bias 0.97) was present and there was no asymmetry on visual inspection of the Begg's funnel plot (not shown).

### Quantitative data synthesis

#### GP IIb/IIIa inhibitors

**Figure 2:**
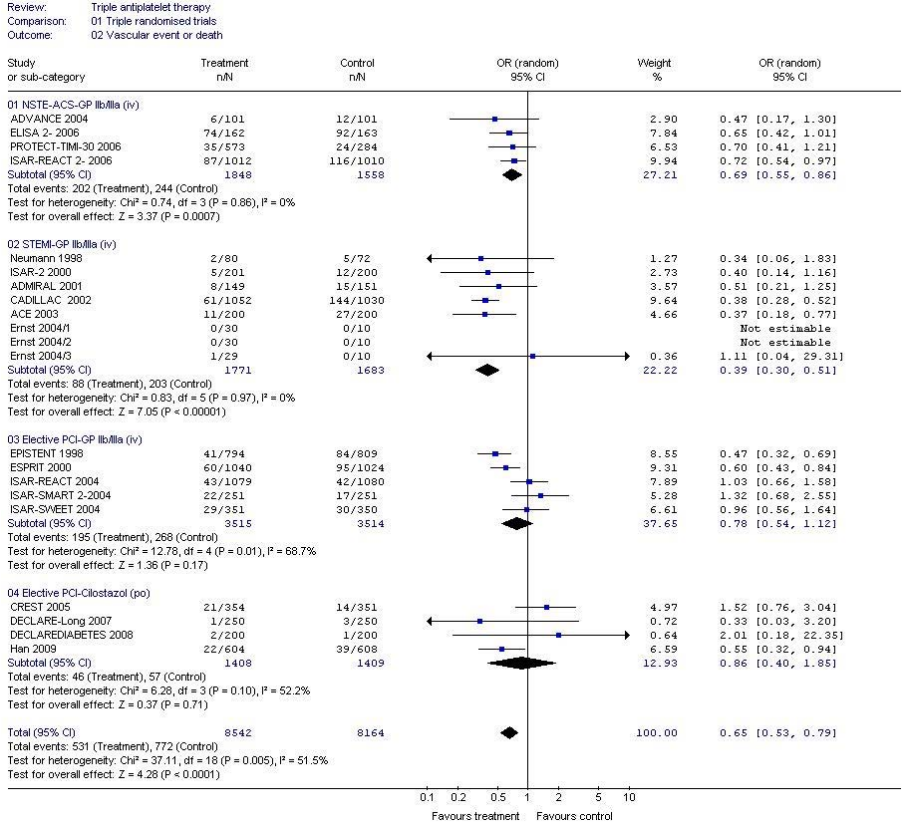
**The effect of triple antiplatelet therapy on vascular event or death**.

**Figure 3:**
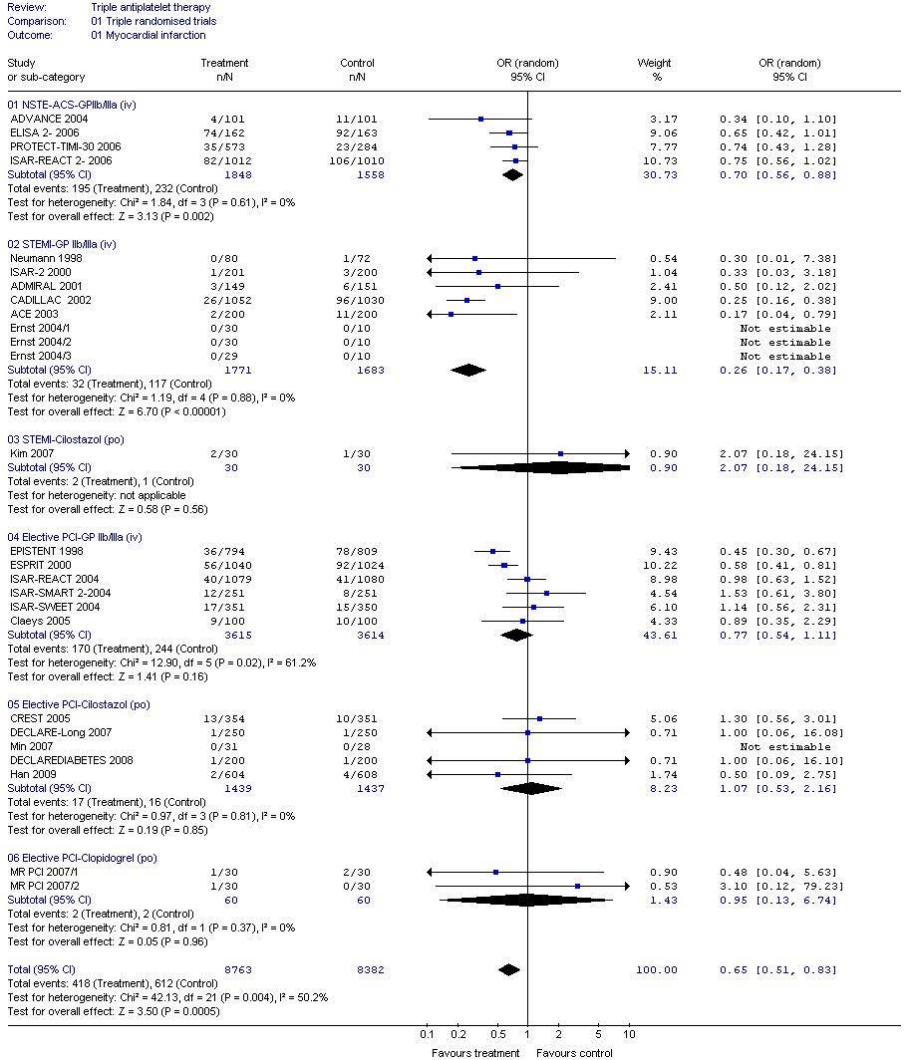
**The effect of triple antiplatelet therapy on myocardial infarction**.

**Figure 4:**
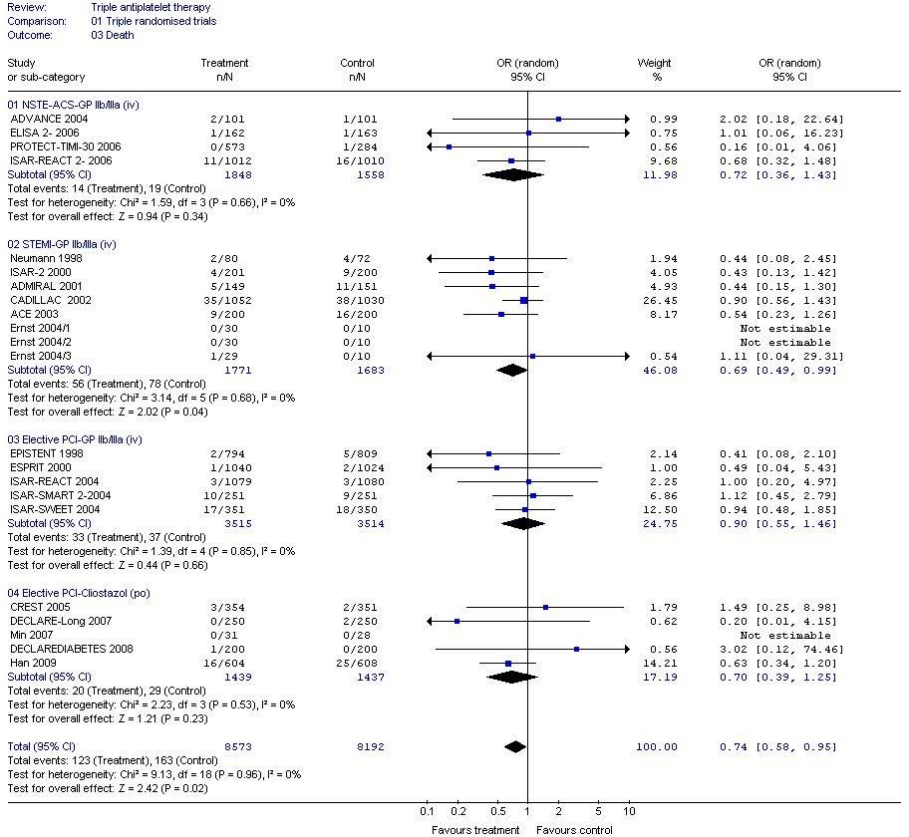
**The effect of triple antiplatelet therapy on death**.

A 30% reduction in composite vascular events (non fatal stroke, non-fatal myocardial infarction or death) and MI alone were seen when GP IIb/IIIa inhibitors were added to dual antiplatelet therapy in patients with NSTE-ACS (Table 2, Figures 2 and 3); a similar magnitude reduction in death was also present although this was non-significant due to the small number of events. Similarly, vascular events and MI were reduced by 60%-70% in patients with STEMI with the addition of GP IIb/IIIa inhibitors; death was also reduced by 30% (Table 2, Figure 4). GPIIb/IIIa inhibitors were significantly more effective in preventing vascular events and MI in patients with STEMI than NSTEMI (chi-square = 10.7, 1 df, *P *<0.01; chi-square = 18.8, 1 df, *P *<0.001, respectively). Trends to reduced vascular events; MI and death were present for GPIIb/IIIa based triple therapy in patients having elective PCI. Only six ischaemic strokes were recorded as an outcome and these came from just four studies; when assessed there was no difference in ischaemic stroke between triple and dual antiplatelet therapy (OR 1.74, 95% CI 0.35-8.65, *P *= 0.5, I^2 ^= 0%).

**Table 2:** Summary of efficacy for triple antiplatelet therapy in randomised controlled trials (significant effects are in bold).

Patients	Intervention	Myocardial infarction			Composite vascular event			Death		
		Triple events/total	Control events/total	OR* (95% CI)	Triple events/total	Control events/total	OR* (95% CI)	Triple events/total	Control events/total	OR* (95% CI)
NSTE-ACS	GP IIb/IIIa	195/1848	232/1558	**0.70 (0.56-0.88)**	202/1848	244/1558	**0.69 (0.55-0.86)**	14/1848	19/1558	0.72 (0.36-1.43)
STEMI	GP IIb/IIIa	32/1771	117/1683	**0.26 (0.17-0.38)**	88/1771	203/1683	**0.39 (0.30-0.51)**	56/1771	78/1683	**0.69 (0.49-0.99)**
	Cilostazol	2/30	1/30	2.07 (0.18-24.15)						
Elective PCI	GP IIb/IIIa	170/3615	244/3614	0.77 (0.54-1.11)	195/3515	268/3514	0.78 (0.54-1.12)	33/3515	37/3514	0.90 (0.55-1.46)
	Cilostazol	17/1439	16/1437	1.07(0.53-2.16)	46/1408	57/1409	0.86(0.40-1.85)	20/1439	29/1437	0.70(0.39-1.25)
	Clopidogrel	2/60	2/60	0.95 (0.13-6.74)						

*Odds ratios (OR) were calculated from random effect models.

CI, confidence interval; NSTE-ACS, non-ST elevation acute coronary syndromes; STEMI, ST elevation myocardial infarction; PCI, percutaneous coronary intervention.

#### Other agents

Neither clopidogrel nor cilostazol, when added to dual antiplatelet therapy (based either aspirin + clopidogrel or aspirin + GP IIb/IIIa inhibitor), had significant effects on vascular events in patients with STEMI or having elective PCI (Table 2). However, the number of trials and patients in the comparisons was relatively small.

### Adverse events

A significant increase in minor bleeding (and trend to increase in major bleeding) was seen with the use of GP IIb/IIIa based triple therapy in patients with STEMI or having PCI (Table 3). As a result, transfusion needs were increased by 80% with GP IIb/IIIa based triple therapy in elective PCI patients. Thombocytopenia was increased eightfold with GP IIb/IIIb based triple therapy in elective PCI patients (Table 3).

**Table 3:** Summary of adverse events during triple antiplatelet therapy(significant effects are in bold).

Patients	Intervention	Major bleeding			Minor bleeding			Blood transfusions			Thrombocytopenia		
		Triple events/total	Control events/total	OR *(95% CI)	Triple events/total	Control events/total	OR* (95% CI)	Triple events/total	Control events /total	OR* (95% CI)	Triple events/total	Control events/total	OR* (95% CI)
NSTE-ACS	GP IIb/IIIa	34/1275	30/1274	1.15 (0.69-1.91)	46/1113	34/1111	1.37 (0.81-2.33)	45/1174	36/1173	1.27 (0.81-2.00)	8/1012	0/1010	17.1 (0.99-296.69)
STEMI	GP IIb/IIIa	11/255	2/195	1.86 (0.43-8.17)	23/255	7/195	**2.73 (1.15-6.46)**	10/281	15/272	0.63 (0.28-1.44)	11/238	4/181	1.51 (0.48-4.71)
	Cilostazol	0/30	0/30										
Elective PCI	GP IIb/IIIa	42/3314	34/3309	1.29 (0.70-2.36)	99/3314	62/3309	**1.60 (1.16-2.21)**	71/3264	40/3263	**1.79 (1.14-2.79)**	17/2269	0/2250	**8.04 (1.82-35.59)**
	Cilostazol	0/1054	1/1058	0.33 (0.01-8.24)	6/1054	7/1058	0.85(0.28-2.56)				1/450	2/450	0.62 (0.08-5.09)
	Clopidogrel	1/60	0/60	3.10 (0.12-79.29)	1/60	0/60	3.10 (0.12-79.23)						

*Odds ratios were calculated from random effect models.

NSTE-ACS, non-ST elevation acute coronary syndromes; STEMI, ST elevation myocardial infarction; PCI, percutaneous coronary intervention.

### Absolute event rates

**Figure 5:**
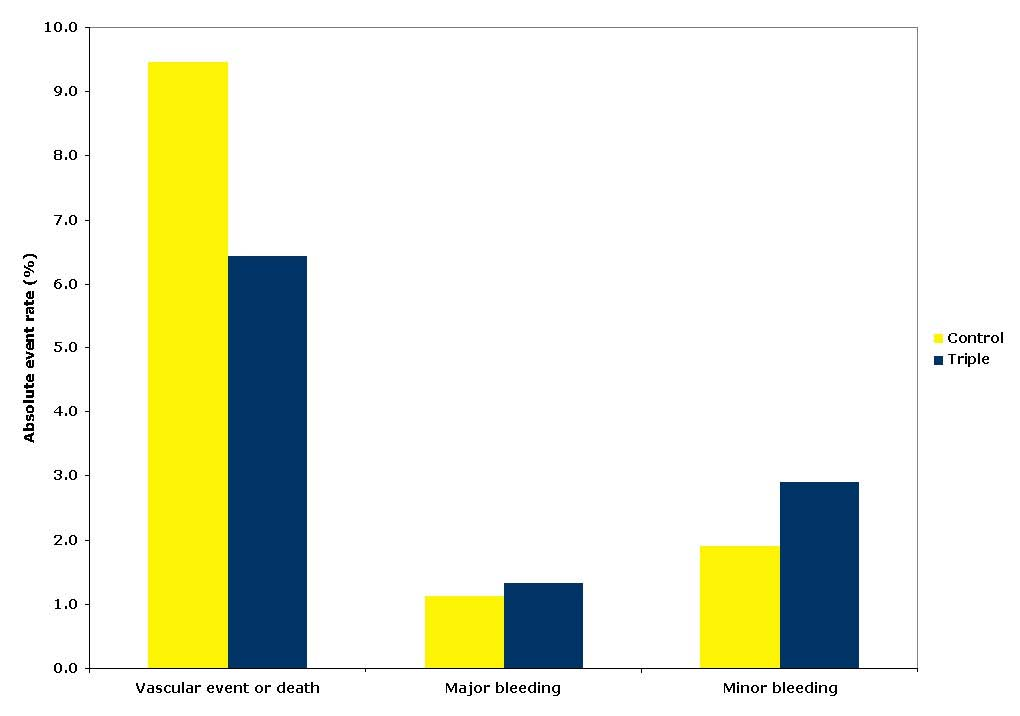
**Absolute event rates for vascular event or death and bleeding**.

Absolute event rates for composite vascular event, major bleeding and minor bleeding are shown in Figure 5. The number of patients benefiting from triple antiplatelet therapy (reduced composite vascular event) was much larger than the number suffering from major bleeding (Figure 5).

## Discussion

The premise of this systematic review was that if two antiplatelet agents are usually superior to one, then three might be better still. Multiple trials were identified which included different groups of patients (NSTE-ACS, STEMI, elective PCI) and add-on drugs (GPIIb/IIIa inhibitors, clopidogrel, cilostazol). The results confirmed that triple antiplatelet therapy involving the addition of an intravenous GPIIb/IIIa receptor antagonist is more effective than dual therapy in reducing vascular events and MI in patients with NSTE-ACS or STEMI. Death was also reduced in those with STEMI. Interestingly, the relative reduction in events appears to be greater in patients with STEMI than NSTE-ACS. A non-significant trend towards a reduction in vascular outcomes was also found in elective PCI patients when treated with a GPIIb/IIIa receptor antagonist, a finding also reported in one other systematic review[24]. In contrast, triple antiplatelet therapy based on oral cilostazol had no effect on vascular outcomes in STEMI and elective PCI patients. Similar results were noted for clopidogrel based triple therapy although this assessment was based on few patients and may reflect a type II error.

Previous meta-analysis involving the addition of iv GPIIb/IIIa inhibitors in the setting of STEMI has revealed conflicting results. One meta-analysis involving six STEMI trials showed no significant reduction in death or recurrent MI[36]. Another showed abciximab as an adjunctive therapy to STEMI patients undergoing stent implantation (but not balloon angioplasty) reduced mortality and recurrent MI[37]. A third meta-analysis involving patients with STEMI undergoing primary angioplasty found that, combined reduced dose thrombolytic therapy and GPIIb/IIIa inhibitors was not superior to Gp IIb-IIIa inhibitors alone[38]. A fourth meta-analysis involving five STEMI trials showed that the use of abciximab in primary stenting may reduce death or MI in patients without preprocedural thienopyridine therapy (but not in those who received thienopyridines)[39]. The present meta-analysis revealed that the addition of iv GPIIb/IIIa inhibitors in the treatment of STEMI patients reduces death, MI and composite vascular outcome. However, the analysis did not include the effects of GPIIb/IIIa inhibitors among different revascularization therapies of STEMI patients.

Unsurprisingly, bleeding events tended to be higher in patients receiving triple antiplatelet therapy although only the analyses involving a GPIIb/IIIa receptor antagonist were associated with significant increases in minor bleeding, this being present in patients with STEMI and elective PCI. Supporting this observation was a need for more blood transfusions in elective PCI. Additionally, treatment with a GPIIb/IIIa receptor antagonist was also associated with an increased risk of thrombocytopenia, again in patients having PCI. However, the balance between treatment efficacy and hazard remains favourable for triple antiplatelet therapy as shown by changes in the absolute event rates for composite vascular event and major bleeding.

Importantly, the duration of randomised treatment was short (12-48 h) in most trials apart from four cilostazol trials where patients received treatment for 6 months (Table 1). Further, trial follow-up was also short varying between 1-12 months. Hence, the superiority of intravenous GPIIb/IIIa receptor antagonist based triple antiplatelet therapy should only be considered for short-term treatment. Also important is that most of the trials gave concomitant heparin to all patients (amounting to quadruple antithrombotic therapy in patients receiving three antiplatelets), which may have contributed to increased bleeding rates. Heparin might also explain the increased rate of thrombocytopenia although the treatment duration was short while thrombocytopenia rates, but not heparin administration, differed between the treatment groups. In the present analysis methodological quality of the trials were assessed in relation to method of randomization and concealment of allocation. Other important factors such as blinding and loss to follow-up that could also influence methodological quality were not assessed in the present analysis.

Whilst the results are clear for patients with STEMI, NSTE-ACS and PCI, there were only very limited data on stroke outcome events and it is not clear whether triple antiplatelet therapy is beneficial among stroke patients. The search process identified one trial of triple antiplatelet therapy performed in 17 patients with chronic ischaemic stroke or TIA (not included in this analysis as it compared triple antiplatelet therapy with aspirin based monotherapy)[40]. Hence, the role of triple therapy in patients with ischaemic cerebrovascular disease cannot be commented on. No trials comparing triple versus dual antiplatelet therapy in patients with PVD was found.

## Conclusions

Triple antiplatelet therapy based on iv GPIIb/IIIa inhibitors was more effective than aspirin-based dual therapy in reducing vascular events, MI and death in patients with acute coronary syndromes (STEMI and NSTEMI). A significant increase in minor bleeding complications was observed among STEMI and elective PCI patients treated with a GP IIb/IIIa based triple therapy. In patients undergoing elective PCI, triple therapy had no beneficial effect and was associated with an 80% increase in transfusions and an eightfold increase in thrombocytopenia. Hence, the use of this enhanced platelet strategy depends on the patient population. The balance between benefit and hazard in patients treated for NSTE-ACS and STEMI lay in favour of giving three antiplatelet agents (typically aspirin, clopidogrel and an intravenous GPIIb/IIIa receptor antagonist) thereby supporting guidelines promoting this approach[19,20]. However, there were no or only few data available for the use of triple antiplatelet therapy for preventing recurrence in patients with chronic IHD, acute or chronic stroke or peripheral artery disease. Further research is now required to assess the role of triple antiplatelet therapy in such patients.

## Abbreviations

IHD: ischaemic heart disease; iv: intravenous; MI: myocardial infarction; NSTE-ACS: non-ST elevation acute coronary syndrome; PCI: percutaneous coronary intervention; PVD: peripheral vascular disease; STEMI: ST elevation myocardial infarction; TIA: transient ischaemic attack; VE: vascular events.

## Competing interests

CG and RW have no competing interests. PB is the Chief Investigator of two trials of triple antiplatelet therapy for stroke prevention, one completed and one ongoing http://www.tardistrial.org/[40].

## Authors' contributions

All authors contributed equally to this work. CG was involved with searches for studies, input of data, analysis and writing. RW participated in searches for studies and writing. PB was involved with the design, development of search strategies, analysis and writing. All authors read and approved the final manuscript.

## Appendix

Search strategy: MEDLINE (OVID)

01 triple antiplatelet therapy/

02. triple antiplatelet$.tw.

03. triple therapy.tw.

04. combined antiplatelet/

05. combined antiplatelet.tw.

06. aggressive antiplatelet therapy/

07. aggressive antiplatelet therapy.tw.

08. antiplatelet therapy/

09. antiplatelet therapy.tw.

10. 1 or 2 or 3 or 4 or 5 or 6 or 7 or 8 or 9 or 10

11. aspirin/

12. dipyridamole/

13. clopidogrel/

14. ticlopidine/

15. prasugrel/

16. cilostazol/

17. triflusal/

18. glycoprotein IIb/IIIa receptor antagonists/

19. abciximab/

20. tirofiban/

21. eptifibatide/

22. 11 or 12 or 13 or 14 or 15 or 16 or 17 or 18 or 19 or 20 or 21

23. 10 and 22

24. ST elevation myocardial infarction/

25. STEMI.tw.

26. non-ST elevation acute coronary syndrome/

27. NSTEACS.tw.

28. acute coronary syndrome/

29. ACS.tw.

30. stroke/

31. transient ischaemic attack/

32. TIA.tw.

33. acute limb ischaemia/

34. peripheral arterial disease/

35. peripheral vascular disease/

36. PVD.tw.

37. 24 or 25 or 26 or 27 or 28 or 29 or 30 or 31 or 32 or 33 or 34 or 35 or 36

38. 23 and 37

39. randomized controlled trials/

40. randomized-controlled-trial.pt.

41. controlled-clinical-trial.pt.

42. random allocation/

43. double-blind method/

44. single-blind method/

45. 39 or 40 or 41 or 42 or 43 or 44

46. exp clinical trials/

47. clinical-trial.pt.

48. (clin$ adj trial$).ti, ab.

49. ((singl$ or doubl$ or trebl$ or tripl$) adj (blind$)).ti, ab.

50. random$.ti, ab.

51. 46 or 47 or 48 or 49 or 50

52. research design/

53. comparative study/

54. exp evaluation studies/

55. follow-up studies/

56. prospective studies/

57. (control$ or prospective$ or volunteer$).ti, ab.

58. 53 or 54 or 55 or 56 or 57

59. 45 or 51 or 52 or 58

60. 38 and 59

## Pre-publication history

The pre-publication history for this paper can be accessed here:

http://www.biomedcentral.com/1741-7015/8/36/prepub
